# Comparison of a Bioelectrical Impedance Device against the Reference Method Dual Energy X-Ray Absorptiometry and Anthropometry for the Evaluation of Body Composition in Adults

**DOI:** 10.3390/nu10101469

**Published:** 2018-10-10

**Authors:** Kaitlin Day, Alastair Kwok, Alison Evans, Fernanda Mata, Antonio Verdejo-Garcia, Kathryn Hart, Leigh C. Ward, Helen Truby

**Affiliations:** 1 Department of Nutrition, Dietetics and Food, Monash University, Notting Hill, VIC 3168, Australia; alastair.kwok@monash.edu (A.K.); Alison.evans@monash.edu (A.E.); fernandagmata@gmail.com (F.M.); helen.truby@monash.edu (H.T.); 2 School of Psychological Sciences, Monash University, Clayton, VIC 3168, Australia; Antonio.Verdejo@monash.edu; 3 Department of Nutritional Sciences, University of Surrey, Guildford GU2 7XH, UK; k.hart@surrey.ac.uk; 4 School of Chemistry and Molecular Biosciences, The University of Queensland, Brisbane, QLD 4072, Australia; l.ward@uq.edu.au

**Keywords:** body composition, dual X-ray absorptiometry, bioelectrical impedance, validation, anthropometry

## Abstract

This study aimed to compare the use of the bioelectrical impedance device (BIA) seca^®^ mBCA 515 using dual X-ray absorptiometry (DXA) as a reference method, for body composition assessment in adults across the spectrum of body mass indices. It explores the utility of simple anthropometric measures (the waist height ratio (WHtR) and waist circumference (WC)) for the assessment of obesity. In the morning after an overnight fast (10 h), 30 participants underwent a body composition DXA (GE iDXA) scan, BIA (seca 515), and anthropometric measures. Compared to the DXA reference measure, the BIA underestimated fat mass (FM) by 0.32 kg (limits of agreement −3.8 kg, 4.4 kg); overestimated fat free mass (FFM) by 0.43 kg (limits of agreement −8.2 kg, 4.3 kg). Some of the variation was explained by body mass index (BMI), as for FM, the mean difference of the normal range BMI group was smaller than for the overweight/obese group (0.25 kg and 0.35 kg, respectively) with wider limits of agreement (−4.30 kg, 4.81 kg, and −3.61 kg, 4.30 kg, respectively). There were significant differences in visceral adipose tissue (VAT) volume measurements between methods with BIA systematically overestimating VAT compared to DXA. WC was more strongly correlated with DXA FM (rho = 0.90, *p* < 0.001) than WHtR (rho = 0.83, *p* < 0.001). BIA had some agreement with DXA; however, they are not equivalent measures for the range of BMIs explored, with DXA remaining the more informative tool. WC is a useful and simple assessment tool for obesity.

## 1. Introduction

In Australia, 63.4% of adults are overweight or obese, according to their body mass index (kg/m^2^, BMI), [1]. With growing obesity rates comes an increase in lifestyle diseases, such as type 2 diabetes mellitus and cardiovascular disease [2,3]. It is widely accepted that the risk of developing an obesity-related disease is more closely associated with body fat distribution than an individual’s body fat percentage [4]. Central adiposity is of particular concern, which tends to be associated with excess subcutaneous adipose tissue and visceral adipose tissue (VAT) [5]. VAT is a highly metabolically active tissue which stimulates lipolytic activity, increasing circulating levels of free fatty acids. Through this and other mechanisms, it orchestrates a switch from an anti-inflammatory to a pro-inflammatory profile of cytokines in circulating plasma [6,7,8]. This drives metabolic dysregulation, and as such, those with a central deposition of VAT are at a higher risk of several obesity-related diseases which is often independent of overall obesity [9,10,11,12,13]. 

BMI is a widely used method for the assessment of obesity, with a cut-off of ≥25 kg/m^2^ utilized to discriminate individuals of normal weight from overweight and obese individuals [14]. Although BMI is well-accepted at a group level for the assessment of metabolic risk and the risk of premature death, at an individual level, as a surrogate measure of body fatness, it is unable to provide an indication of body fat distribution. Optimal use of other body composition assessment tools depends upon whether the individual is of a normal weight or is overweight, as defined by BMI. Simple anthropometric measures, such as waist circumference (WC) and its ratio with height (WHtR) are used to determine risks associated with central adiposity. The development and validation of WHtR as a tool to identify those at risk of metabolic complications of obesity has been well-established for adults and children [15,16]. 

Assessment of body composition is used in a variety of settings, from weight management clinics to sports performance, and screening at a population level for risk of obesity complications [17]. Therefore, methods for body composition assessment must be able to detect clinically relevant changes quickly and robustly. Owing to the wide range of applications for body composition assessment, an instrument must be accurate and precise for a range of body sizes; or where such an instrument is not available, it must be determined which method of body composition is most suitable for a particular population, as defined by their body size. Consequently, establishing agreement and/or equivalence between different methods is necessary to understand the appropriate use and setting for each instrument. 

Dual X-ray absorptiometry (DXA) is a commonly used method for the assessment of body composition, especially bone density and soft tissue [18]. The GE Lunar iDXA uses a fan beam system to measure bone density and total cell mass, and it can also estimate fat mass (FM) and fat-free mass (FFM). DXA is a well-accepted reference method of body composition assessment (FM and FFM) in adults and children and the GE Lunar iDXA, with its proprietary CoreScan software, also estimates VAT. This measure, although not directly measured, has shown good agreement with computer topography (CT) and magnetic resonance imaging (MRI) [18,19,20]. DXA machines are expensive, large, and are not readily portable. They require specialist staff, and present a radiation hazard, albeit a low one, to participants. This makes them unsuitable as a method for screening large numbers of potentially at-risk patients in the community. 

Bioelectrical impedance devices (BIA) are smaller, some being hand-held, and faster, and require little training for their use. They use a harmless electrical current, rather than radiation, to assess body composition. BIA measures the opposition (impedance or resistance) to the flow of an electric current through the body, and uses this information to predict total body water (TBW). Predictions are made via the use of algorithms, often empirically derived and device-specific, in order to quantify FFM, calculated as TBW/0.732 where 0.732 is an assumed hydration fraction of the FFM. FM is obtained by subtracting FFM from total mass. The seca^®^ mBCA 515 Analyser (seca^®^, Hamburg, Germany) offers the additional calculation of VAT volume through an in-house developed proprietary algorithm [21]. 

Current literature suggests that BIA could be a viable alternative to DXA for determining body composition, with BIA offering an inexpensive, faster, and less invasive option. However, to date, studies have not investigated its utility across a wide spectrum of body sizes (BMI) [22,23]. Shafer et al. (2009) found that BIA significantly overestimated FM and FFM compared to DXA. The magnitude of the overestimation was dependent on BMI [24]. Contrastingly, a study by Anderson et al. (2012) demonstrated good agreement between BIA and DXA for FM and lean body mass in a range of BMIs [25]. Other studies have reported small errors in FM measurements [23,26] and even smaller errors in FFM measurements [27,28] in various BIA analyzers. This highlights that the validity of BIA instruments varies across manufacturers and, as such, each new BIA model should be independently assessed against accepted reference methods, such as DXA. 

There is a clear need for a better understanding of the strengths and weaknesses of different techniques of body composition instruments, which is particularly true when proprietary algorithms are incorporated into simple devices but with little explanation, such as with the seca 515 mBCA Analyzer. Often, BIA devices are used interchangeably with other body composition methods, and so there is a need to explore whether this is appropriate. Other simple measures include WHtR and WC, which are quick and simple anthropometric measures often used to assess central adiposity, and can be surrogate indicators of disease risk in both adults and children [16,29]. The BIA and DXA instruments tested here offer detailed body composition assessment, including the quantification of VAT. With multiple options for the assessment of body composition, it is important to be able to provide evidence for the optimal use of these tools, and to define their accuracy and inter-changeability in terms of body sizes. 

There is a need to quantify VAT, as it is potentially a more precise screening measurement for identifying those at risk of metabolic disorders associated with obesity. The reference methods for the quantification of VAT is MRI or CT scanning. The GE iDXA with CoreScan technology is widely used for body composition assessment, and also reports the quantification of VAT; this has been confirmed against CT scanning [19]. Between BIA devices themselves, variability in agreement with the reference methods of either DXA or MRI have been observed. Few BIA devices give a quantified measurement of VAT, with many reporting risk scores [30,31]. Some BIA devices, such as the seca mBCA 515 Analyzer, offer the ability to measure VAT. In order to gain a greater understanding of the potential benefit of different body composition analyzers as a screening tool, they must be compared across a range of BMIs to assess their appropriate use and determine the strengths and weaknesses of different methods. 

The aims of this study were: to compare the use of the seca mBCA 515 Analyzer for body composition (FM and FFM) against a body composition scan, using the GE Lunar iDXA as a reference method; to explore the VAT measures across both instruments; and to assess the utility of WHtR and WC as indicators of body fatness. 

## 2. Materials and Methods 

### 2.1. Participants

Thirty participants enrolled in this observational study. Recruitment commenced in October 2015 and concluded in September 2016. Participants were recruited through online staff and student forums and poster advertisements at Monash University, Melbourne, Australia. Inclusion criteria were: healthy adults, who were able to comprehend and consent to the study in English; being aged between 18 and 65 years; having a BMI between 18.5 kg/m^2^ and 50 kg/m^2^; and those who reported to have a stable weight and were not actively attempting to lose or gain weight. Exclusion criteria included: pregnant women; anyone who had exposure to radiation three months prior to study enrolment; and those with standard exclusions for DXA and/or BIA, such as those with implanted defibrillator devices or prostheses. 

Participants were required to attend one three-hour session in the morning after an overnight fast. All subjects gave their full informed consent for inclusion before participation in the study. The study was conducted in accordance with the Declaration of Helsinki, and the protocol and supporting documents were approved by the Monash University Human Research Ethics Committee (CF15/2790-2015001139).

### 2.2. Anthropometric Measurements

Height was measured using a Holtain fixed stadiometer (Holtain Ltd., Crosswell, Wales, UK) to the nearest 0.1 cm [32]. Weight was measured using stand-on digital scales (seca, Hamburg, Germany) to the nearest 0.1 kg. Anatomical WC was measured at the mid-point between the top of the iliac crest and the bottom of the lowest rib, to the nearest 0.5 cm [33]. All measurements were repeated twice using standard operating procedures, and all assessors were trained by a single level 2 certified anthropometrist. WHtR was calculated as WC divided by height, and BMI was calculated as body weight (kg) divided by height, squared (m^2^).

### 2.3. Dual-Energy X-Ray Absorptiometry

The DXA scan was conducted on a GE Lunar iDXA (GE Healthcare, Software Lunar DPX enCORE 2012 version 14.0, Madison, WI, USA). Each participant received a total body scan, conducted by a single qualified radiographer (AE) who was experienced in DXA scanning. Participants were scanned in the supine position and VAT was estimated using the android region (from the ribs to the iliac crest) via the inbuilt CoreScan software (GE Healthcare, Software Lunar DPX enCORE 2012 version 14.0, Madison, WI, USA). FFM was determined by the sum of bone mineral content and lean soft tissue values, and FM was also determined. The coefficient of the variations were 0.74% for FM, 0.48% for lean mass, and 11.84% for VAT volume. 

### 2.4. Bioelectrical Impedance Analysis

The seca mBCA 515 (seca, Hamburg, Germany) uses multi-frequency 8-point stand-on bioelectrical impedance analysis to measure TBW by applying an electrical current of 100 µA to the body. The drop in voltage between sensor electrodes at the hands and feet is used to determine total body water. The manufacturer’s operating instructions and proprietary software calculated FM, FFM, and VAT volume from total body water, weight, WC, height, age, and gender [21]. The BIA device measures at 20 frequencies, ranging from 1 kHz to 1000 kHz. Participants were scanned once in the standing position, with four electrodes at the feet and four electrodes at the hands. Participants were instructed to remain stationary for the duration of the scan, which lasted 60 s. 

### 2.5. Statistical Analysis

All statistics were analyzed using IBM SPSS Statistics 24 (IBM, New York, NY, USA). The sample was analyzed as a whole group and then split into those with a weight within the normal range (BMI < 18.5 kg/m^2^ and <25 kg/m^2^) and those with a weight in the overweight or obese range (BMI ≥ 25 kg/m^2^), as defined by BMI. Descriptive characteristics are displayed as means ± the standard deviation for the total sample, both males and females. For FM, FFM, and VAT, one-sample *t*-tests were performed to initially assess the variation from zero for the difference between methods. If the variation was not significantly different from zero, a Bland-Altman plot was created to assess the agreement between methods and determine the limits of agreement for each variable [34]. Linear regression was used to determine the proportional bias in differences between the methods. Two one-sided T tests (TOST) were used to determine clinically significant equivalence between BIA and DXA measures of body composition [35]. The delta was set to 5% of the average, as measured by DXA, the reference method in this instance. These data were not normally distributed; therefore, correlations between BIA, DXA, and WHtR were analyzed using Spearman’s Rank correlation. Concordance between DXA and BIA measurements of FM, FMM, and VAT were assessed using Lin’s concordance [36]. All graphs were created using GraphPad Prism 7.01 (GraphPad Software, La Jolla, CA, USA).

## 3. Results

Descriptive characteristics of the population are displayed in Table 1. The average age of the sample population was 29.9 ± 11.2 years, and the average BMI was 29.2 ± 7.3 kg/m^2^ with a range from 18.8 kg/m^2^ to 48.9 kg/m^2^, where 73% were of Caucasian descent. Of the total sample, 33% had a BMI in the normal weight range (*n* = 10), 47% were classified as overweight (*n* = 14), and 20% were obese (*n* = 6), as defined by the World Health Organization’s cutpoints for BMI. Height (cm) and weight (kg) were significantly different between males and females (Table 1). 

Table 2 displays FM, FFM, and VAT, as measured by the DXA and BIA, and Table 3 displays the corresponding correlations between BIA, DXA, WHtR, and WC for FM, FFM, and VAT. FM and VAT measures were significantly correlated for the total sample. WC and BIA FFM (kg) were significantly correlated (rho 0.972, *p* < 0.001), as well as BIA FFM (kg) and WHtR (rho 0.500, *p* < 0.01). There was a strong correlation between BIA and DXA for FM (kg), (rho 0.981, *p* < 0.001) and between WC and BIA VAT (cm^3^) (rho 0.944, *p* < 0.001). For each variable, BIA and DXA were more strongly correlated with WC than WHtR; in the normal weight group, there were no significant relationships between these variables (Table 3). For the overweight/obese group, all measures were significantly correlated for FM and VAT. For FFM, BIA was significantly correlated with WC and WHtR (rho 0.823, *p* < 0.001 and rho 0.470, *p* < 0.05, respectively). Again, in this group, BIA and DXA were more strongly correlated with WC than WHtR for all measures. Concordance was strong between BIA and DXA for FM and FFM (0.992 and 0.947, respectively); however, concordance was poor for VAT measured by BIA and DXA (−0.016).

**Figure 1:**
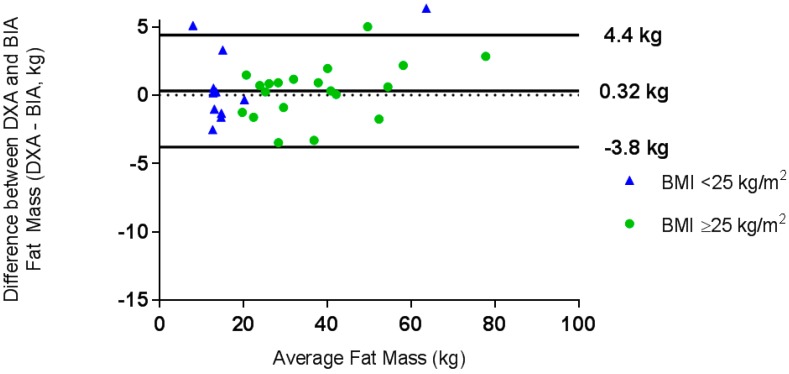
Bland-Altman plot of the difference between fat mass, as measured by dual energy X-ray absorptiometry (DXA) and bioelectrical impedance (BIA), against the mean fat mass (kg) for the whole group. ●  BMI ≥ 25 kg/m^2^, ▲ BMI < 25 kg/m^2^.

**Figure 2:**
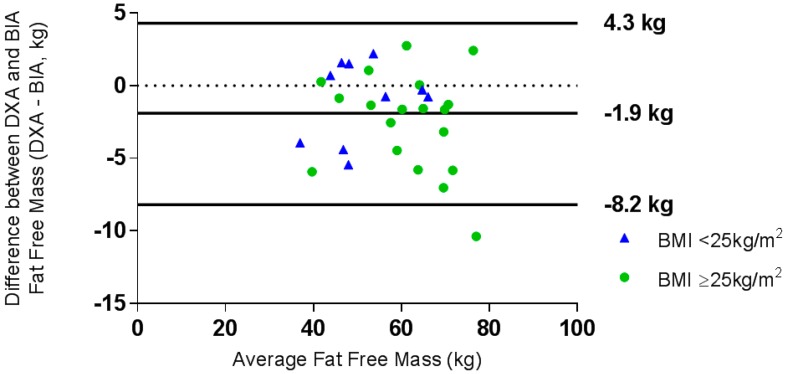
Bland-Altman plot of the difference between fat-free mass (kg), as measured by DXA and BIA, against the mean fat-free mass (kg) for the whole group. ● BMI ≥ 25 kg/m^2^, ▲ BMI < 25 kg/m^2^.

A one-sample *t*-test demonstrated that the difference between methods for VAT measurement was significantly different from zero (*t* = −6.4, *p* < 0.001) for the total sample and when the sample was split into normal weight and overweight/obese groups; therefore, it was not appropriate to construct Bland-Altman plots for this variable. Linear regression analysis showed that BIA significantly overestimated VAT compared to DXA (*t* = −13.0, *p* < 0.001). A Bland-Altman plot determined the level of agreement for FM measurements (Figure 1) and FFM measurements (Figure 2). 

BIA underestimated FM by 0.32 kg (limits of agreement −3.77 kg, 4.40 kg, ±27.6%) compared with DXA. For the normal weight group, BIA underestimated FM by 0.25 kg (limits of agreement: −4.30 kg, 4.81 kg, ±34.4%) compared with DXA, and for the overweight and obese group, BIA underestimated fat mass by 0.35 kg (limits of agreement: −3.61 kg, 4.30 kg, ±25.4%) compared with DXA. There was good agreement between the methods for FM, and whilst the mean difference was smaller for the normal weight group, the limits of agreement were narrower for the overweight and obese group (Figure 1). 

Regression analysis showed that there was no proportional bias between measures of FM or FFM for the whole group or either of the normal weight or overweight/obese groups (data not shown). BIA overestimated FFM by 0.43 kg, and whilst the limits of agreement were larger, this represented a smaller proportion of mean FFM (−8.2 kg, 4.3 kg, ±21.7%) than for FM (Figure 2). The mean difference between the methods for FFM was larger for the normal weight group, as were the limits of agreement compared to the overweight/obese group (Figure 2). Clinically acceptable equivalence (5%) was not demonstrated for both FM and FFM for the total sample, nor when split into normal weight and overweight groups. Equivalence was demonstrated at 25.8% for FM and 11% for FFM for the whole group.

## 4. Discussion

This study aimed to better understand the strengths and weaknesses of two relatively new instruments that purport to measure body composition, as well as VAT in adults across the spectrum of the BMI range. Both devices, the GE iDXA and seca BIA instruments, incorporate proprietary algorithms into simple-to-use instruments, but with little explanation as to their measurement derivation. A strength of the seca 515 BIA is its non-invasive nature and ease of use; however, our results indicate that the seca BIA device showed greater agreement with DXA for FM measurements, especially in individuals with larger amounts of FM. The two methods did not demonstrate clinically relevant equivalence for measures of FM or FFM; this highlights that different devices for body composition are not interchangeable.

VAT volume is an independent risk factor for a host of lifestyle-related diseases [9,10]. Quantification achievable in a clinical setting could help to prioritize patients for intervention and further characterize their risk. Finding an inexpensive and faster alternative to MRI or CT scans for the quantification of VAT volume remains a challenge. GE iDXA and seca BIA may provide practical alternatives to quantify VAT, albeit that the seca BIA systematically and significantly overestimated VAT compared to the iDXA. Pietiläinen et al. (2013) found other impedance devices have also shown poor agreement between BIA and DXA for assessment for VAT, and the specially designed BIA device, ViScan, has limited VAT prediction ability [18,37]. Previous studies have shown good agreement between DXA and MRI, or CT measures of VAT [19,38]. Concordance was also found to be low, suggesting that the two methods were not measuring the same parameter. Indeed, it is difficult to see how segmental impedance analysis, in which the trunk is measured as a whole rather than the visceral compartment per se, can specifically predict VAT. Impedance techniques with a focus on measuring the impedance of the central abdominal region would appear to hold more promise, although current devices have not shown good clinical performance to date [39,40]. Alternatively, the seca mBCA 515 Analyzer demonstrates good agreement with DXA for the quantification of total body FM, itself a helpful indicator of lifestyle disease risk, independent of VAT [41]. 

Waist circumference is a valuable tool in assessing central adiposity and, as such, this simple anthropometric measure should not be overlooked, as it is an informative indicator of obesity and risk of related diseases [42,43]. Although anthropometric measures were found to be significantly correlated with DXA measurements of FM and VAT (especially for the overweight and obese participants), anthropometry and BIA may not be an informative measure of body composition for individuals within the normal weight range, due to the smaller proportion of FM seen in this group. Thus, DXA may be preferred to BIA or anthropometry for body composition analysis in normal-weight individuals.

The limitations of this study were the small sample size, especially in the normal weight group, with the majority of the participants being of Caucasian descent; and not being able to draw conclusions on the generalizability of the instruments for use in body composition assessment across other ethnic groups, due to body composition variability observed between ethnicities [44]. However, the large range of BMIs explored in this study was a strength, as it allowed assessment of agreement and equivalence of measures across a wide range of body sizes. Future work should focus on validating the seca mBCA 515 Analyzer in a larger sample with a range of BMI and different ethnicities.

The limits of agreement were larger than those previously reported [25,28], with the overweight and obese group showing narrower limits of agreement than the normal weight group. These differences in mean error and limits of agreement could be attributed to a range of factors, including: (1) the use of different proprietary algorithms by each device, and; (2) the large range of BMIs explored in this study. These findings strongly suggest that for consistency, any repeated or longitudinal measures on any individual should be performed using the same device or technique.

WC and WHtR are quick, simple, and cheap methods of assessing body fatness [45]. WC is an especially good indicator of body fatness for individuals who are overweight or obese, but cannot provide information on body composition. In busy clinic settings, WC should not be overlooked as a quick and simple assessment of a patient’s risk of obesity. BIA may be able to provide more detailed information on body composition than anthropometry, and has demonstrated good agreement with DXA for FM, particularly for those who are overweight or obese, as defined by BMI. However, the seca mBCA 515 Analyzer and DXA are not equivalent techniques for the range of BMIs explored in this study; thus, DXA remains more informative for body composition than the seca mBCA 515 Analyzer.

## Figures and Tables

**Table 1:** Participant characteristics (mean ± standard deviation).

	Male (*n* = 14)	Female (*n* = 16)	Total (*n* = 30)
**Age (years)**	32.3 ± 12.9	27.9 ± 9.4	29.9 ± 11.2
**Height (cm)**	177.7± 3.6 *	170.0 ± 8.9 *	173.6 ± 7.9
**Weight (kg)**	99.7 ± 26.9 **	79.9 ± 25.4 **	89.1 ± 27.6
**BMI (kg/m^2^)**	31.6 ± 8.4	27.2 ± 5.8	29.2 ± 7.3
**Range**			18.8–48.9 ^a^
**Anatomical waist circumference (cm)**	105.9 ± 23.4 (*n* = 13)	94.1 ± 26.4	99.4 ± 25.4
**Waist to height ratio**	0.6 ± 0.1	0.6 ± 0.1	0.6 ± 0.2

* *p* < 0.05, ** *p* < 0.01 for differences between males and females by independent samples *t*-test. ^a^ Body mass index (BMI) range, number of participants is stated as (*n*=) for parameters that vary from total sample.

**Table 2:** Fat mass, fat-free mass, and visceral adipose tissue volume, as measured by DXA and BIA (normal weight BMI < 25 kg/m^2^ and overweight and obese BMI ≥ 25 kg/m^2^).

	DXA			BIA		
	Normal Weight Group (*n* = 10, 6 = Female)	Overweight and Obese Group (*n* = 20, 10 = Female)	Whole Group (*n* = 30)	Normal Weight Group (*n* = 10, 6 = Female)	Overweight and Obese Group (*n* = 20, 10 = Female)	Whole Group (*n* = 30)
**Fat mass (kg) Mean (SD)**	26.60 (15.08)	31.15 (18.00)	29.63 (16.96)	27.23 (14.24)	30.36 (17.77)	29.32 (16.49)
**Fat free mass (kg) Mean (SD)**	57.70 (9.30)	57.62 (12.86)	57.65 (11.63)	58.92 (10.47)	58.88 (12.69)	58.90 (11.78)
**Visceral adipose tissue (litres) Mean (SD)**	1.25 (1.47)	1.04 (1.13)	1.11 (1.23)	0.81 (0.98)	5.24 (5.19)	3.76 (4.74)

Data presented as mean values (standard deviation). DXA: dual energy X-ray absorptiometry; BIA: bioelectrical impedance.

**Table 3:** Correlation matrix (Spearman’s rho) between BIA, DXA, WC, and WHtR for fat-free mass, fat mass, and visceral adipose tissue (normal weight BMI < 25 kg/m^2^, overweight and obese ≥ 25 kg/m^2^).

	Normal Weight Group (*n* = 10)	Overweight and Obese Group (*n* = 20)	Whole Group (*n* = 30)
**Fat free mass**			
WHtR vs. BIA	0.418	0.470 *	0.500 **
WHtR vs. DXA	0.365	−0.095	0.073
WC vs. BIA	0.620	0.823 ***	0.972 ***
WC vs. DXA	0.277	0.147	0.222
BIA vs. DXA	−0.292	0.425	0.323
**Fat mass**			
WHtR vs. BIA	0.552	0.716 ***	0.815 ***
WHtR vs. DXA	0.588	0.747 ***	0.828 ***
WC vs. BIA	0.444	0.815 ***	0.888 ***
WC vs. DXA	0.571	0.836 ***	0.901 ***
BIA vs. DXA	0.588	0.989 ***	0.981 ***
**Visceral adipose tissue**			
WHtR vs. BIA	0.409	0.870 ***	0.822 ***
WHtR vs. DXA	0.091	0.653 ***	0.743 ***
WC vs. BIA	0.573	0.987 ***	0.944 ***
WC vs. DXA	0.103	0.807 ***	0.880 ***
BIA vs. DXA	0.323	0.826 ***	0.869 ***

Data presented as rho values. * *p* < 0.05; ** *p* < 0.01; *** *p* < 0.001. WHtR: waist to height ratio; WC: anatomical waist circumference.
